# Characteristics of Tuberculosis Cases that Started Outbreaks in the United States, 2002–2011

**DOI:** 10.3201/eid2103.141475

**Published:** 2015-03

**Authors:** Maryam B. Haddad, Kiren Mitruka, John E. Oeltmann, Emma B. Johns, Thomas R. Navin

**Affiliations:** Centers for Disease Control and Prevention, Atlanta, Georgia, USA (M.B. Haddad, K. Mitruka, J.E. Oeltmann, E.B. Johns, T.R. Navin);; Emory University, Atlanta (M.B. Haddad, E.B. Johns)

**Keywords:** tuberculosis, disease outbreaks, disease transmission, infectious, United States, bacteria, tuberculosis and other mycobacteria, Mycobacterium tuberculosis, incarceration, drug use, homelessness

## Abstract

A review of 26 tuberculosis outbreaks in the United States (2002–2011) showed that initial source case-patients had long infectious periods (median 10 months) and were characterized by substance abuse, incarceration, and homelessness. Improved timeliness of diagnosis and thorough contact investigations for such cases may reduce the risk for outbreaks.

Tuberculosis (TB) is an airborne disease caused by the bacterium *Mycobacterium tuberculosis* that usually affects the lungs. Most TB cases do not start outbreaks. Contact investigations undertaken after a person receives a diagnosis of infectious TB ideally should identify and treat infected contacts before the infection progresses to disease (*1*). Genotyping data in the United States provide reassurance that most cases do not result from recent transmission (*2*).

However, when TB outbreaks do occur, they can put tremendous strain on local public health resources (*3**,**4*). A necessary component of all outbreaks is that they must begin with a source case. Recognizing the characteristics of such patients soon after the TB diagnosis could help focus interventions to interrupt transmission and reduce the risk for an outbreak (*5*). We describe a nonrandom sample of TB source cases that started outbreaks in the United States.

## The Study

We identified outbreak source cases through a review of investigation reports of TB outbreaks written by Centers for Disease Control and Prevention (CDC) staff during 2002–2011. In a previous publication, we described how CDC teams that assist public health partners with TB outbreaks write standardized reports about field investigations, which include primary data collection and patient re-interviews (*3*). For this review, we included US-based outbreaks that 1) had >3 culture-confirmed cases that had epidemiologic links and TB strains with matching genotypes and 2) had the initial source case in the transmission chain, as ascertained by that investigation, identified in the written report. At least 2 co-authors independently reviewed each outbreak report to abstract patient demographic, clinical, and social characteristics. When this dual data entry process showed discrepancies, the authors met to review the report and achieve consensus.

Our main interest was the initial source case; that is, the TB case that began a chain of *M. tuberculosis* transmission that would become locally recognized as an outbreak. Outbreak duration was calculated beginning on the treatment start date for the first reported case and continuing through the treatment start date for the last case as noted at the time of the investigation. The infectious period for pulmonary TB cases was assumed to begin 3 months before TB symptom onset and to end with the initiation of TB treatment (*1*).

Of the 65 TB outbreaks that CDC helped investigate during 2002–2011, a total of 26 met the inclusion criteria. The most common reason for exclusion was not meeting the threshold of >3 culture-confirmed cases that had epidemiologic links and matching genotypes (19 outbreaks); 11 adequately large outbreaks or longstanding genotype clusters were excluded because an initial source case could not be unambiguously identified. Other reasons for exclusion included an international setting (6 outbreaks) or missing data (3 outbreaks).

In 20 of the 26 TB outbreaks, the source case-patient was also the first patient in the outbreak to come to the attention of public health authorities (i.e., also the index case-patient). In the other 6 outbreaks, a delay in the diagnosis of the source case meant that another outbreak case was identified before the source case (median delay 2 months, maximum 7 months).

Characteristics of the 26 source cases are provided in the Table. Case-patients ranged in age from 18 to 62 years; most were US-born men, and the distribution of race/ethnicity was similar to that seen in national TB surveillance (*6*). Case-patients had long infectious periods (median 10 months, range 3−36 months); after seeking medical attention for TB symptoms, these patients often experienced delays in TB diagnosis and thus delays in treatment initiation. All patients had pulmonary TB that was smear positive for acid-fast bacilli. Most patients reported excess alcohol or illicit drug use; half had been incarcerated at some point in the past, and nearly half had been homeless in the year before diagnosis. Most cases came to public health attention because the patient sought care for TB symptoms rather than through contact investigations.

**Table:** Characteristics of source case-patients for 26 investigated tuberculosis outbreaks, United States, 2002–2011

Characteristic	No. (%)
Demographics	
US-born	19 (73)
Male sex	23 (88)
Race/ethnicity	
White non-Hispanic	7 (27)
Black non-Hispanic	13 (50)
Hispanic	6 (23)
Clinical and laboratory characteristics	
Sputum smear positive for acid-fast bacilli	26 (100)
Cavitary tuberculosis on chest radiograph	21 (81)
HIV co-infection	2 (8*)
*Mycobacterium tuberculosis* lineage†	
EuroAmerican	18 (69)
East Asian	4 (15)
Social risk factors for tuberculosis	
Excess alcohol use	16 (62)
Illicit drug use	14 (54)
Homelessness within previous year	11 (42)
Incarceration at diagnosis	4 (15)
Incarceration ever	13 (50)
Reasons for prolonged infectious period‡	
Delay in seeking care after symptom onset	8 (31)
Delayed diagnosis once sought care	15 (58)
Noncompliance during treatment	7 (27)
Method of case detection	
Self-reported symptoms led to diagnosis	21 (81)
Tuberculosis contact investigation	1 (4)
Other screening	1 (4)
Unknown	3 (12)


*Of 24 patients for whom HIV test results were available. †Genotype lineage was not determined for 4 outbreaks that occurred in 2002−2003, before spoligotyping was routine. ‡Causes not always documented and not mutually exclusive.

Of the 26 outbreaks, 9 were limited to 1 generation of spread from the source case-patient to that person’s direct contacts; the other 17 cases had further waves of transmission beyond the source case-patient. At the time of the investigations, median outbreak duration was 13 months (range 4–151 months), and a total of 242 cases (median 8 per outbreak) were diagnosed. The 4 outbreaks in which the source case-patient was incarcerated at diagnosis ranged in size from 7 to 9 cases. For the 11 outbreaks in which the source case-patient had recently been homeless, the median outbreak size at time of investigation was 9 cases, with outbreak size ranging from 3 to 27, the latter being the largest outbreak in our review.

## Conclusions

In this nonrandom sample of 26 TB outbreaks in the United States during 2002–2011, we found that characteristics common among TB cases that started outbreaks included pulmonary TB smear-positive for acid-fast bacilli, patient substance abuse, and prolonged infectious periods. The largest outbreaks involved source case-patients who were incarcerated or had been homeless.

Persons with TB can spread the disease until it is correctly diagnosed and treated (*7*). In TB control, the focus is typically on the individual patient, health care provider, and public health factors that contributed to a delayed diagnosis, inadequate isolation or treatment, or otherwise suboptimal response to an individual TB case (*8*). As the frequency of TB cases continues to decline in the United States, however, so does provider experience with its diagnosis, which raises the possibility that the recent trend toward more cases of pulmonary TB being diagnosed in later disease stages might be a related consequence (*9*).

When contact investigations are incomplete because of limited resources or hard-to-reach populations, TB outbreaks can spread (*3**,**4*). Substance abuse, incarceration, and homelessness, social risk factors that are common among the TB source cases in our review but that greatly complicate contact investigations, have been shown to increase the likelihood of genotype cluster growth (*10**,**11*) and outbreak development (*3*).

This retrospective review of secondary data sources has several limitations. The beginning of an outbreak can be difficult to determine; the concept of an initial source case is an artificial construct if one considers that every source must have had its own progenitor. In addition, the definition of the outbreak’s duration and size was determined on the basis of the timing of the CDC field investigation. The generalizability of the characteristics of these source cases is uncertain. 

However, a strength of this analysis is that a group of experienced TB investigators considered all the information available to determine which of the known cases involved in an outbreak most likely represented the source. An additional advantage over many population-based genotype studies is that epidemiologic links among patients were ascertained, enabling confirmation that cases in the same genotype cluster were indeed part of the same chain of transmission. We also knew direction of transmission, enabling us to establish source cases without having to make assumptions on the basis of the timing of diagnoses (*12*). In addition, we had information from patient medical records about TB symptom onset, which enabled us to examine variables related to infectious periods and delayed diagnosis.

TB contact investigations for all persons diagnosed with pulmonary TB with acid-fast bacilli smear-positive test results are a well-known public health priority (*1*). This review underscores the particular importance of prompt and thorough investigations for TB cases confirmed by positive smear for acid-fast bacilli in which patients have experienced substance abuse, incarceration, or homelessness. Public health departments should work with local health care providers to address barriers to accessing care faced by marginalized populations and in recognizing and diagnosing TB once symptomatic patients seek medical attention. 
